# Fingertip wearables and obstructive sleep apnea: is event level precision the key?

**DOI:** 10.1093/sleep/zsaf026

**Published:** 2025-01-30

**Authors:** Steven Holfinger, M Jeffery Mador

**Affiliations:** Department of Internal Medicine, Division of Pulmonary, Critical Care and Sleep Medicine, Davis Heart and Lung Institute, College of Medicine, The Ohio State University Wexner Medical Center, Columbus, OH, USA; Division of Pulmonary, Critical Care and Sleep Medicine, University at Buffalo, Buffalo, NY, USA

Wearable home sleep testing solutions have exploded onto the scene in recent years, aiming to improve obstructive sleep apnea (OSA) detection with improved patient comfort, reduced costs, and more accessible multi-night testing. These new technologies can generally be grouped by the use of photoplethysmography (PPG), the technology underlying pulse oximetry and peripheral arterial tonometry (PAT) signals, with other methods of identifying OSA without PPG including tracheal sounds (AcuPebble, BresoDX1), mandibular movements (Sunrise), or respiratory effort (Wesper Lab) [1]. While PPG has been available for decades, OSA detection was not possible until recently due to excessive signal noise. The initial PAT devices (WatchPAT) improved PPG signal quality mechanically using a probe to create an even pressure field around the finger; however, more recent PAT approaches attempt to remove noise from the signal computationally (NightOwl, Somfit, ANNE Sleep). While not identifying as a device using PAT, the Belun ring uses deep-learning neural networks to assess the PPG for OSA detection [1]. Other unique Software as a Medical Device (SaMD) approaches include the SleepImage SaMD, which uses rule-based algorithms to derive cardiopulmonary coupling from the PPG, and EnsoSleep PPG, a SaMD capable of scoring respiratory events and 4-stage sleep using approved PPG devices with pulse oximetry.

In this issue of SLEEP, Chen et al. introduce a fingertip-worn PPG-based TipTraQ device to predict OSA [2]. Similar to predicate devices using PPG, the TipTraQ leverages artificial intelligence (AI) with a cloud-based 1-dimensional convolutional neural network (CNN) for OSA detection. TipTraQ also uses pulse rate and accelerometry to estimate total sleep time using a 1-dimensional CNN. The algorithms were initially developed and tested in 240 Asian adults, with ground-truth comparisons against polysomnography scored using the 4% desaturation criteria for hypopnea scoring. External performance evaluation was also reported in a similar Asian adult population with 112 participants, using different recording equipment, scoring software, and participants from a separate hospital.

The TipTraQ analysis included many of the typical metrics to measure performance by including OSA classification by group (the confusion matrix), the receiver operator curve (ROC) and sensitivity and specificity to detect OSA using AHI cutoffs ≥ 5 and ≥15, measures of correlation with AHI using root mean square error (RMSE) and Pearson, Spearman, and intraclass correlation coefficients (PCC, SCC, ICC), and Bland-Altman plots for limits of agreement. The results of the TipTraQ were comparable to predicate devices, and the authors added another form of analysis by conducting event-level assessments of event detection accuracy. They defined a method of assessing individual event detection accuracy by looking only at the end-of-events; if a predicted event ends within a prespecified window (10 s) of a ground-truth event, it is considered positive. An alternative method for event-level analysis recently published in SLEEP can be seen in Choi et al., where true-positive events were defined by the overlap of a predicted event with a ground-truth event [3].

While a high degree of event-level accuracy is certainly preferable, the importance of this analysis in wearable home testing devices is not yet clear. Individual event detection during polysomnography is useful for identifying positional OSA, REM-related OSA, and inter-event trends such as periodic breathing as occurs with Cheyne-Stokes respiration. For the TipTraQ, position and sleep stages are not yet reportable, nor are central events, and the utility of the hypnogram on the report is yet to be seen. When manual review by a clinician involves overscoring of events, higher event accuracy would result in fewer event corrections and improved efficiency. However, the current iteration of the TipTraQ does not include manual overscoring. While manual overscoring is available on most of the novel OSA detection technologies, it is not clear if human manual scoring actually improves accuracy over the AI scoring solutions. The ability to provide an event-by-event analysis would allow us to answer this question. When considering that the primary use of these devices is to identify moderate-to-severe OSA with an AHI ≥ 15, the TipTraQ excels, achieving an impressive ROC curve area of 0.98. However, like other similar devices, TipTraQ faces challenges with accurately detecting mild sleep apnea or confirming its absence (Table 3 of Chen et al.) [2]. When mild sleep apnea is detected by the device, there is a 6%–11% chance of the subject not having sleep apnea (4/63 in the internal validation set and 3/28 in the external validation set) and an 18-22% of the subject having moderate-to-severe sleep apnea (14/63 in the internal validation set and 5/28 in the external validation set). In subjects in whom the device does not detect sleep apnea, there was a 13%–28% chance of their having mild sleep apnea (11/39 in the derivation set and 4/31 in the validation set).

While the initial results are promising, the TipTraQ has several limitations. Snoring, position, and sleep stages have not yet reported. The algorithm has not yet been validated outside of the Taiwanese population, limiting its application for widespread use. In addition to the lack of discrimination between obstructive and central events, the study inclusion criteria removed those more likely to have central sleep apnea, including those with New York Heart Association (NYHA) classification III–IV heart failure, severe strokes, or using opioids. The choice of using the 4% hypopnea criteria alone, without the use of 3% and arousals to determine hypopneas, also limits application for clinicians using the currently recommended AASM scoring guidelines [4]. In addition, the participants studied had relatively few comorbidities. Of the 32/100 with hypertension, diabetes mellitus, or COPD in the validation set, the model performed significantly worse (*p* = 0.0077) compared to those without these comorbidities in the secondary analysis (Appendix of Chen et al.) [2]. Thus, the results in this study may represent optimal performance of the device and may more closely approximate comparable devices in a real world setting.

Individual event analysis may open the door for improved patient care. When providers can review events and the logic is known, provider confidence in AI accuracy can increase adoption rates. The burgeoning field of explainable AI could be a solution that balances the principles of transparency, interpretability, and explainability. Yet, while understanding the mechanism behind AI identification of events is preferable, the most accurate models for sleep-disordered breathing may transcend human comprehension. Correct identification of sleep-disordered breathing diagnoses will be challenging as long as the AHI remains the gold standard outcome for OSA classification, however, because developed models will continue to inherit the limitations of the AHI being a simplified outcome measure. Ideally, features of individual events might be used to predict OSA endotypes or guide treatment options with improved training and outcome datasets. Alternative approaches to determine OSA severity and predict the cardiovascular and neurocognitive sequelae have been under evaluation, including promising findings when using the hypoxic burden, which is now reported by EnsoSleep PPG [5, 6]. Further information or better indices are needed for clinicians to better determine who would best benefit from treatment of mild OSA (AHI 5–15), or identify those at highest risk for long-term adverse outcomes.
